# Effects of intracanal cryotherapy on postoperative pain in necrotic teeth with symptomatic apical periodontitis: a randomized controlled clinical trial

**DOI:** 10.3389/fdmed.2025.1543383

**Published:** 2025-04-14

**Authors:** Muhammad Zubair Ahmad

**Affiliations:** Department of Conservative Dental Sciences, Qassim University College of Dentistry, Buraydha, Saudi Arabia

**Keywords:** apical periodontitis, cold saline, cryotherapy, periapical disease, postoperative pain, pulp necrosis

## Abstract

**Objectives:**

The present study aimed to assess the effects of intracanal cryotherapy on pain following single-visit non-surgical root canal treatment (NSRCT) of molar teeth with pulpal necrosis and symptomatic apical periodontitis (SAP).

**Methods:**

This parallel-two arm, single-blind, randomized superiority clinical trial was registered at www.clincaltrials.gov (NCT05611736). Patients referred for NSRCT meeting the inclusion criteria were included. Preoperative radiographs, pulp sensibility tests, and pain scores on the visual analog scale (VAS) were recorded. Following shaping and cleaning, 302 patients were randomly allocated to the two groups (*n* = 151). In the experimental group, final irrigation was done using 0.9% physiologic saline solution at 2.5 °C, whereas in the control group, final irrigation was done using the same solution at room temperature. All treatments were performed in a single visit. Analgesics intake and presence, duration, and intensity of pain using the VAS at 6, 24, 72 h, and 1 week were recorded. Any adverse events were recorded. Data was analyzed using the Mann–Whitney *U* test and the Student's *t* test (*P* < 5%).

**Results:**

Patients in the cryotherapy group had significantly less postoperative pain at 6, 24, and 72 h (*P* < 0.05). There was no difference in postoperative pain at 1 week (*P* > 0.05). No adverse event was recorded in either group during or immediately after root canal treatment.

**Conclusion:**

Cryotherapy significantly reduces postoperative pain in single-visit root canal treatment of molars with pulp necrosis and SAP. It can be considered a biocompatible, economical, and straightforward method for managing postoperative pain.

**Clinical Trial Registration:**

www.clincaltrials.gov, identifier (NCT05611736).

## 1 Introduction

Pain control is essential for effective patient management (1). Painful stimuli initially activate the sympathetic nervous system (SNS) through a process involving nociceptor signaling and autonomic responses (2). Various endogenous inflammatory mediators sensitize and activate nociceptors, contributing to pain perception. This activation typically occurs in response to tissue injury or damage and is a fundamental aspect of the body's innate immune response (3). Pain following root canal treatment is a significant health concern that can impact quality of life in the short term and, in some cases, over the long term (4). In endodontic practice, managing postoperative pain is a critical consideration. Postoperative pain is particularly likely in teeth with necrotic pulp, symptomatic apical periodontitis, and preoperative pain (5). Patients may experience varying levels of pain before, during, and after endodontic treatment. According to Sathorn et al. (6), postoperative endodontic pain can range from 3% to 58%. Pain management is one of the primary objectives of endodontic treatment for necrotic teeth with symptomatic apical periodontitis.

Postoperative endodontic pain management has been investigated extensively. Various strategies have been proposed to control postoperative pain, such as a detailed explanation of the procedure before initiating endodontic treatment and patient calming approaches (7), applying different kinematics and mechanical techniques during root canal instrumentation (8), occlusal reductions (9), preparation of glide path (10), anesthesia of longer duration (11), and medications such as nonsteroidal anti-inflammatory drugs (12), antihistamines (13), acetaminophen (14), salicylic acid (15), narcotic analgesics (16), combinations of salicylic acid with narcotic analgesics (17), and steroidal anti-inflammatory drugs (1, 18).

Cryotherapy decreases the temperature of tissues for therapeutic reasons. The term is derived from the Greek words “cryos” which means “very cold” and “therapeia” which means “cure” (19). As early as 3,000 BCE, the ancient Egyptians were the first to use cold to treat injuries and reduce inflammation. However, in medical literature, James Arnott first reported the application of cold in malignant diseases by applying ice and salt (20). Cryotherapy has been one of the treatment options to manage pain since the 1960s (21). A decrease in metabolic activity, inhibition of neural receptors, and a decrease in local blood flow are three of the fundamental physiological responses of the tissues when cold temperatures are applied (22). Root canal irrigation with cold saline reduces the temperature locally, which in turn may cause a reduction in inflammation in the adjacent periradicular tissues (19, 23).

Adverse events and complications may arise because of the cold application and the irrigation of root canals (24). Mitchell et al. compared two different irrigation systems in their *in vitro* study and reported that the extrusion of irrigants ranged from 8.3% to 58.3% (25). However, no clinical study has yet been conducted on the effects of intra-canal cryotherapy application on postoperative pain and associated complications or adverse events in molar teeth with pulp necrosis and symptomatic apical periodontitis. Therefore, the purpose of this study was to assess the effects of intracanal cryotherapy application on postoperative pain in molar teeth with pulp necrosis and symptomatic apical periodontitis and any resulting complications. The null hypothesis of this trial was that there was no significant difference in postoperative pain between intracanal cryotherapy and irrigation at room temperature.

## 2 Materials and methods

### 2.1 Study design

**Figure 1:**
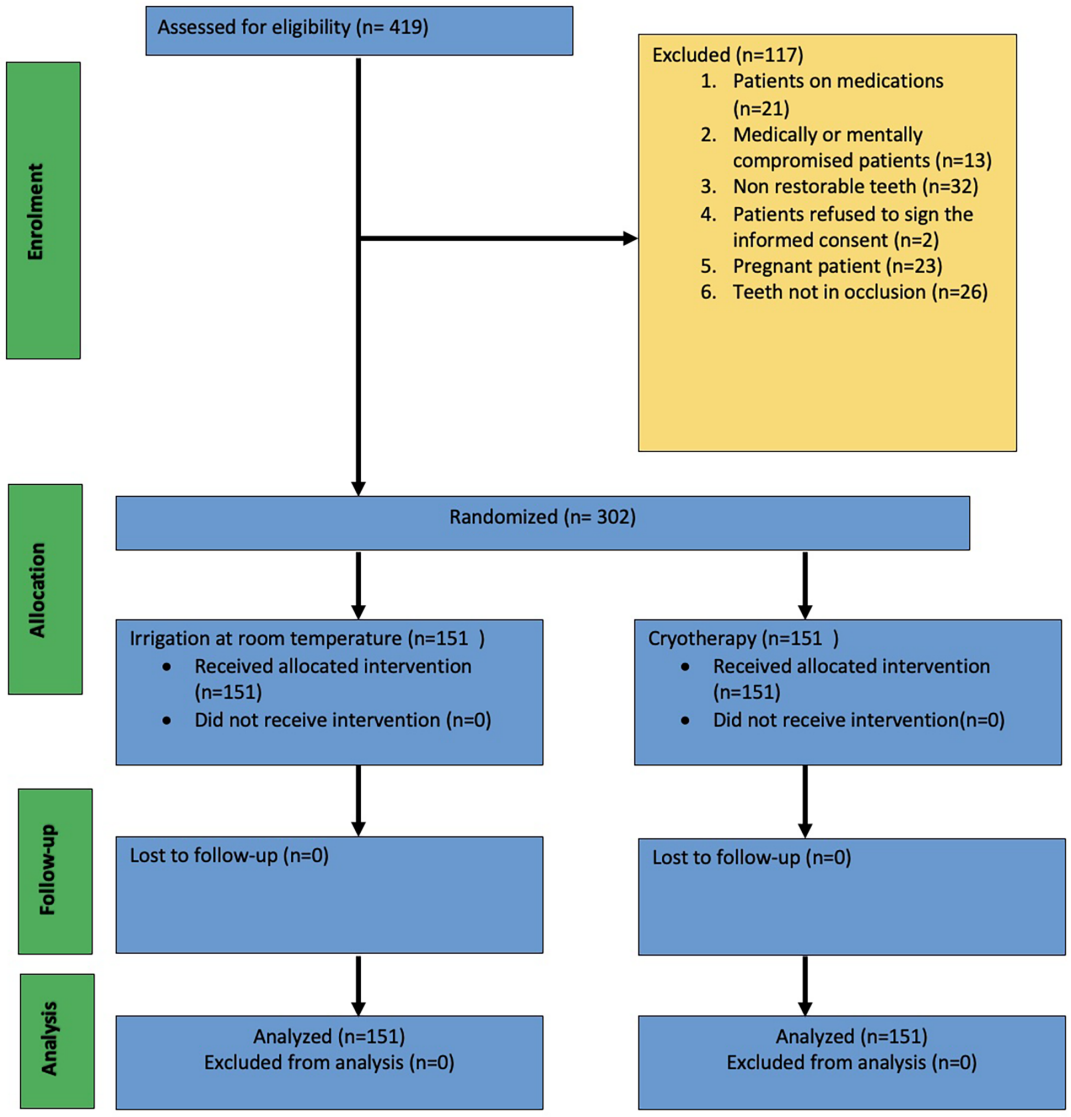
CONSORT flowchart [Adapted with permission from Schulz et al., (26)].

We designed a prospective randomized clinical trial following the ethical principles (including the World Medical Association Declaration of Helsinki). The study protocol was approved by the Institutional Review Board (IRB) of Qassim University, Saudi Arabia (registration no. 21-19-08) and registered with the clinical trials website (http://www.clinicaltrials.gov) with the number NCT05611736. This randomized clinical trial was conducted following CONSORT guidelines (Figure 1) (26). Sample size calculation and power analysis were based on information from a previous study (27). The overall mean and standard deviation (SD) values for pain scores (Visual Analog Scale) in control and cryotherapy groups were 2.01 ± 1.47 and 0.77 ± 1.45 respectively. Using these values, the effect size (Cohen's *d*) was calculated as:

$$d\;=\frac{M_{1}-\;M_{2}}{SD_{\mathrm{pooled}}}$$

whereas the pooled standard deviation was computed as:

$$SD_{\mathrm{pooled}}=\mathrm{sqrt}\left(\frac{\left(SD_{1}^{2}+SD_{2}^{2}\right)}{2}\right)$$

To ensure greater precision in effect estimation and enhance the reliability of our findings, we adopted a conservative approach by increasing the sample size. Similar studies have also utilized larger sample sizes to achieve robust statistical power and generalizability (27, 28). Applying these calculations, we obtained an effect size of 0.85, with an alpha error of 0.05. We considered 128 samples per group to identify meaningful differences between the experimental and control groups. Additional calculations recommended a total adjusted sample size of 151 per group, accounting for a 15% dropout rate. Given this sample size, the statistical power was approximately 0.99, ensuring a high probability of detecting a true effect while maintaining robustness and generalizability in our results. 302 molar teeth with pulp necrosis and symptomatic apical periodontitis had root canal treatments done by one expert operator with more than ten years of experience as a subject specialist. All treatments were completed in a single appointment. All study participants who underwent root canal procedures gave verbal and written consent.

### 2.2 Patient selection and allocation

419 patients who complained of pain in their molar teeth were referred for endodontic treatment in the Alrass dental clinics at the College of Dentistry, Qassim University, Saudi Arabia. Prior to their enrollment, written consent was obtained from each patient, and they were all informed of the study's objectives and design.

Before starting the treatment, palpation and percussion tests were done, and pulpal sensibility was assessed with EndoIce (Hygenic Corp, Akron, OH, USA). The study only included patients with symptomatic apical periodontitis related to molar teeth diagnosed with necrotic pulp (negative thermal stimulation with EndoIce confirmed with an absence of bleeding during access cavity preparation).

The patients recorded their preoperative pain levels by filling out a questionnaire that included a visual analog scale (VAS) score (0–10, with 0 indicating no pain at all and 10 the worst pain). We included only those patients who registered their pain levels as 8, 9, or 10.

We excluded the following cases: pregnancy, endodontic retreatment, patients on pain medications, immunocompromised patients, extremely curved root canals, non-restorable teeth, patients who did not give consent for treatment, root resorption (internal or external), teeth not in occlusion, immature apices of the teeth, teeth with pus-filled canals that could not be dried after shaping and cleaning procedures in one visit, and third molars. We also excluded the patients who did not completely fill out the forms.

We included 302 patients who met the inclusion criteria in this study. Patients were assigned either to experimental or control groups by generating a list of random numbers (https://www.random.org) and stored in an Excel spreadsheet by the assistant staff. Consecutive numbers were assigned to the patients who fulfilled the study inclusion criteria and were willing to participate. The allocation process was concealed by using sequentially numbered, opaque, sealed envelopes containing treatment assignment cards prepared prior to the trial. If a patient had only one tooth eligible for the study, the treatment was assigned according to the randomization. If a patient had two eligible teeth, the first tooth received the randomized treatment, and the second tooth was subsequently treated with the alternative method. For patients with more than two eligible teeth, the treatment for the third tooth and beyond was determined by randomization. The operator did not open the envelope containing the treatment allocation. The list of assignments remained confidential until the analyses were completed. An assistant opened the envelopes and double-checked the list to ensure that the patient would be assigned to the correct group. Following the shaping procedure, the assistant gave the clinician the information. Preoperative tooth-related parameters, including tooth location, occlusal contacts, and the presence or absence of radiolucent lesions, as well as patient-related characteristics, including age and sex, were recorded.

### 2.3 Endodontic treatment procedure

Root canal treatment procedures were performed in a single visit. We anesthetized the patients with two cartridges of anesthetic containing 4% articaine HCl with 1:100,000 epinephrine (Ultracaine D-S Forte; Aventis, Istanbul, Turkey). Intraligamental articaine 4% was injected for cases requiring supplemental anesthesia. Rubber dam isolation was used to complete all of the procedures. The access cavity was prepared using the new, sterile round bur (Diatech, Coltene Whaledent) with water as coolant. A conventional straight-line access was achieved. A size 10K-file (Dentsply Maillefer, Ballaigues, Switzerland) was inserted into the canal. We used the Root ZX mini apex locator (Morita Corp., Kyoto, Japan) to establish the working length, which was confirmed by taking a periapical radiograph. The size 10K-file was used as a patency file. We flushed the root canals using 5.25% NaOCl. A smooth glide path was formed using the ProGlider instrument (Dentsply Sirona) without lubrication agents. The ProGlider instrument was operated using X-Smart Plus (Dentsply Sirona, Ballaigues, Switzerland) endodontic motor following manufacturer instructions (16:1 contra angle, 5 Ncm, 300 rpm). Upon resistance, the instrument was withdrawn, the canal recapitulated using size 10K-file, and irrigated using 5.25% NaOCl. The procedure was repeated until ProGlider passively reached the working length.

Root canals were prepared using Protaper Gold (Dentsply Tulsa Dental Specialties, Tulsa, OK, USA) files with X-Smart Plus (Dentsply Sirona, Ballaigues, Switzerland) endodontic motor. The files were operated in continuous rotation motion using brushing movements following manufacturer instructions. Copious irrigation was done using 5.25% sodium hypochlorite (NaOCl) solution using 30-G side-perforated closed-ended needle (NaviTip, Ultradent, South Jordan, UT, USA) at a rate of 5 ml/min. A 10 ml of irrigant was used for each canal. After changing each instrument, we confirmed the patency of the canal using a size 10K-file. We flushed the root canals for 1 min using 5 ml of 17% EDTA solution. The irrigants were agitated using EndoActivator (Dentsply Tulsa Dental Specialties, Tulsa, OK, USA) for three cycles of 20 s each, with irrigant renewal at the beginning of each 20-sec cycle. The root canals were dried using sterile paper points.

### 2.4 Experimental group (*n* = 151)

In the experimental group, a final root canal irrigation was performed with 0.9% physiologic saline solution at 2.5°C using a 30-G side-perforated closed-ended needle (NaviTip, Ultradent, South Jordan, UT, USA) positioned 2 mm shorter than the working length. The irrigant was stored in the refrigerator until use.

### 2.5 Control group (*n* = 151)

In the control group, a final root canal irrigation was performed with 0.9% physiologic saline solution at room temperature using a 30-G side-perforated closed-ended needle (NaviTip, Ultradent, South Jordan, UT, USA) positioned 2 mm shorter than the working length.

Because of temperature differences in irrigant-containing syringes, the operator could not be blinded. However, patients were not aware of the intervention assigned as well as temperature of irrigant, hence they were kept blinded from the assigned groups. We used sterile paper points to dry the canals in both groups, and final obturation was performed using a cold lateral condensation technique using gutta-percha cones and AH Plus® (Dentsply Maillefer, Ballaigues, Switzerland) sealer. The fit of the master cone and the quality of the canal obturation were confirmed using periapical radiographs. Subsequently, the access cavities were temporarily restored with glass ionomer material (Riva Light Cure, Southern Dental Industries-SDI, Victoria, Australia).

The postoperative pain experienced by patients was documented using the Visual Analog Scale (VAS). The patients indicated their pain level by marking a point on a 10-cm continuous line, with endpoints representing “no pain” and “severe/unbearable pain.” The distance between the patient's mark and the point representing no pain was measured and recorded. Patients were given the form containing the VAS following the completion of the endodontic treatment. They submitted the completed forms during their second appointment, which was scheduled seven days after completion of the treatment. The pain was categorized based on severity: 0 = No pain, 1–3 = Mild pain, 4–6 = Moderate pain, and 7–10 = Severe pain. The patients were instructed to complete the form at 6, 24, 72 h, and one week. Patients were contacted daily for three days to remind them to record their pain levels. Patients were also asked to record the duration of pain. In case of severe, unbearable pain, patients were informed to take oral analgesics. Ibuprofen 600 mg/8–12 h was recommended as rescue medication. Patients were told to contact the operator for any emergency relevant to the teeth under endodontic treatment.

The distribution of the data was tested using the Kolmogorov–Smirnov test. The chi-square test was used to test the difference among the categorical variables. Comparisons between groups were computed using Student's *t*-test and Mann–Whitney *U* tests for parametric and nonparametric data, respectively. The data was analyzed using IBM SPSS 28.0 software (IBM Corp, Armonk, NY, USA) at a 5% level of significance.

## 3 Results

Table 1 shows the distribution of baseline demographic and clinical data in both treatment groups. A total of 302 molar root canals were done, with 151 in each group. The mean age in the cryotherapy group was 43.70 years, and the mean age in the control group was 39.87 years (*p* = 0.921). The mean preoperative pain scores in cryotherapy and control groups were 8.20 ± 0.4 and 8.21 ± 0.41, respectively (*p* = 0.775). There was no statistically significant difference between teeth locations (*p* = 0.386). No adverse event occurred in any group during or immediately after the treatment. All patients included in this study returned the VAS forms. Only three patients took the analgesic medication once, 12 h postoperatively. All were female patients in the control group.

**Table 1:** Distribution of baseline demographic and clinical data in both pools of patients (chi-square test).

Baseline features	Control *n* (%)	Cryotherapy *n* (%)	Total	*P* value
Sex				
Male	86 (48.86%)	90 (51.14%)	176	0.641
Female	65 (51.59%)	61 (48.41%)	126	
Location				
Maxillary	107 (51.69%)	100 (48.31%)	207	0.386
Mandibular	44 (46.32%)	51 (53.68%)	95	
Periapical radiolucency				
No	71 (52.21%)	65 (47.79%)	136	0.488
Yes	80 (48.19%)	86 (51.81%)	166	
Age group				
<30	38 (60.32%)	25 (39.68%)	63	0.077
30–50	85 (50%)	85 (50%)	170	
>50	28 (40.58%)	41 (59.42%)	69	

The mean postoperative pain scores in cryotherapy and control groups were 0.47 and 3.33, respectively (*p* < 0.001). Patients in the cryotherapy group reported significantly lower VAS scores at 6, 24, and 72 h postoperatively (*p* < 0.001). No patient reported the presence of pain at one week in either group. The intensity and incidence of postoperative pain are shown in Tables 2, 3. The results of descriptive statistics showed that the cryotherapy group had lower values for postoperative pain at 6-h (cryotherapy group *Mdn* = 0, *Control* group *Mdn* = 3), 24-h (cryotherapy group *Mdn* = 0, *Control* group *Mdn* = 2) and 72-h (cryotherapy group *Mdn* = 0, *Control* group *Mdn* = 2). There was no difference between both groups at one week (cryotherapy group *Mdn* = 0, *Control* group *Mdn* = 0). A Mann–Whitney *U* test showed significantly less pain in the cryotherapy group at 6-h (*U* = 1,854, *p* = <.001, *r* = .77), 24-h (*U* = 2,040.5, *p* = <.001, *r* = .75) and, 72-h (*U* = 1,787.5, *p* = <.001, *r* = .79). There was no difference between both groups at one week (*p* = 1).

**Table 2:** Results for incidence and intensity of pain (chi-square test).

Incidence	Intensity	Control	Cryotherapy	*P* value
No		17	113	<0.001*
Yes	Mild	99	38	<0.001*
	Moderate	29	0	
	Severe	6	0	

* Statistically significant value.

**Table 3:** Results of postoperative pain levels after 6, 24, 72 hours, and 1 week (Mann–Whitney *U*-test).

Variable	Mean ± SD	Median	Quartile 1	Quartile 3	Interquartile range	*U*	*z*	*P* value	*r*
6 h								<.001*	0.77**
Cryotherapy	0.47 ± 0.87	0	0	0.5	0.5	1,854	—		
Control	3.33 ± 1.77	3	3	3	0		13.42		
24 h						2,040.5		<.001*	0.75**
Cryotherapy	0.34 ± 0.64	0	0	0.5	0.5		—		
Control	2.42 ± 1.2	2	2	3	1		13.02		
72 h						1,787.5	−13.8	<.001*	0.79**
Cryotherapy	0.09 ± 0.29	0	0	0	0				
Control	1.47 ± 0.77	2	1	2	1				
1 week									
Cryotherapy	0	0	0	0	0	11,400.5	NaN	1	NaN
Control	0	0	0	0	0				

NaN, not an applicable number.

* Statistically significant value.

** Large effect size.

There was no significant difference between postoperative pain and gender in either group (*P* > 0.05). Similarly, tooth location did not significantly affect postoperative pain in either group (*P* > 0.05).

## 4 Discussion

This prospective randomized controlled clinical trial aimed to investigate whether cryotherapy may effectively decrease postoperative pain in patients who require endodontic treatment in molar teeth due to preoperative pain, pulp necrosis, and symptomatic apical periodontitis. Postoperative pain management is considered among the most important objectives of endodontic treatment (29). The cold saline solution as the final intracanal irrigant may reduce the temperature to greater than 10 °C on the external root surface, maintaining this low temperature for up to 5 min, which may be sufficient to produce a local anti-inflammatory effect in periradicular tissues (23).

The results of this study indicate that postoperative pain is significantly reduced when root canals were irrigated with cold, sterile saline solution at 2.5 °C for 5 min, compared to the pain levels observed in the control group of patients. Therefore, the null hypothesis was rejected. The perception of postoperative pain is influenced by multiple factors, making it difficult to study the impact of just one factor. Preoperative pain indicates prior injury to the periradicular area, and it is a significant predictor of more frequent and intense postoperative pain (30), which is the reason for including patients with preoperative pain scores of 8 and above. The intervention strategies during the procedure may be crucial for predicting and alleviating postoperative pain (1, 30, 31). Effects of cold saline irrigation have been studied previously by researchers in single-visit root canal treatment of teeth with vital pulps (28) and in multi-visit root canal treatment of uniradicular teeth with necrotic pulps (27). So far, no study has reported the effects of cold saline irrigation during single-visit root canal treatment on postoperative pain in molar teeth with pulp necrosis and symptomatic apical periodontitis. This study assessed the effects of cold saline irrigation during the single-visit root canal treatment of necrotic molar teeth and evaluated the incidence and severity of postoperative pain, frequency of rescue medications intake, and adverse events.

Sathorn et al. (32) in their systematic review reported a 6.3% higher healing rate in single-visit root canal treatment of necrotic teeth with symptomatic apical periodontitis as compared to multi-visit root canal treatment. It is historicaly advised that experienced practitioners may prefer one-visit endodontics (33). In the present study, an experienced endodontist performed the pre-established treatment protocol for all patients. We confirmed the pulp necrosis with the absence of bleeding from the pulp following access cavity preparation. All attempts were made to eliminate the other factors that may affect pain perception, such as medically compromised patients and patients on medications. The overall postoperative pain levels experienced by patients in this study were low to moderate. Only 6 (1.99%) patients reported severe postoperative pain. 29 (9.6%) patients reported moderate postoperative pain. 138 (45.7%) patients reported mild postoperative pain. None of the patients in the cryotherapy group experienced moderate or severe levels of pain. Overall, 43% of the patients reported no pain at 6-h and 24-h postoperatively. 51% of the patients reported no pain 72-h postoperatively. None of the patients reported pain at one week postoperative follow-up.

Applying thermal treatments, whether hot or cold, to tissues can lead to variations in blood flow. These alterations in blood flow have the potential to either stimulate or inhibit nociceptors, thereby causing corresponding increases or decreases in metabolic activity. Prior research has demonstrated that cryotherapy applications effectively reduce bleeding, inflammation, muscle spasms, musculoskeletal pain, connective tissue regression, and nerve conduction velocity (22, 34). A systematic review found that after acute soft tissue injuries, cryotherapy is effective in reducing short-term pain and inflammation (35). The reduction in pain and inflammation observed after cryotherapy can be attributed to mechanisms such as vasoconstriction, decreased biochemical reactions, and a slowdown in cellular metabolism (19).

Furthermore, subsequent hypoxia-related lesions and tissue damage can be avoided using cryotherapy. Additionally, vasoconstriction helps to prevent the development of edema (36). In a systematic review, Sadaf et al. (37) concluded that intracanal cryotherapy is effective in significant reduction of postoperative pain at 6-h and 24-h in teeth with pulpal or periradicular pathosis.

Vera et al. (27) reported a significant reduction of postoperative pain levels at 6-h, 24-h, and 72-h of intracanal cryotherapy after multi-visit root canal treatment in uniradicular necrotic teeth. Keskin et al. (28) reported a significant reduction in postoperative pain levels at 24-h and 48-h of intracanal cryotherapy after single-visit root canal treatment in vital teeth. Jain et al. (38) found a significant reduction in postoperative levels of pain in the intracanal cryotherapy group at 6-h, 24-h, and 48-h. Nandhini et al. (39) reported significantly lower postoperative pain levels at 6-h, 12-h, 24-h, 48-h, and four days in the intracanal cryotherapy group as compared to the control group after single-visit root canal treatment in mandibular premolar teeth with acute irreversible pulpitis. However, no significant difference was found at seven days postoperatively. The results of this study are consistent with the aforementioned studies, indicating a significant reduction of postoperative pain in the cryotherapy group after 6-h, 24-h, and 72-h and no difference in cryotherapy and control groups at seven days.

It is important to mention that some studies reported different results related to intracanal cryotherapy during endodontic treatment. Alharthi et al. (40) reported that although there were lower postoperative pain levels at 6-h, 24-h, and 48-h, they found no significant difference between intracanal cryotherapy and the room-temperature irrigation group. Gundogdu and Arslan (41) found significantly lower pain levels in the cryotherapy group on the first, third, fifth, and seventh days. In our study, although the pain levels were significantly lower in the cryotherapy group at 6-h, 24-h, and 72-h, there was no difference between cryotherapy and control groups at one week.

EndoVac (Kerr Endo, Orange County, CA, USA) is a root canal irrigation system that uses negative pressure (42). Because of minimal or no apical extrusion, the use of EndoVac has been reported to reduce the risk of periradicular inflammatory reactions (42, 43). Sadaf et al. (37) conducted a meta-analysis on the effects of intracanal cryotherapy on postoperative pain, and their subgroup analysis revealed no significant difference between the EndoVac and needle syringe irrigation techniques. Research has also indicated that conventional needle irrigation may be associated with significantly greater irrigant extrusion and more postoperative pain compared to negative apical pressure systems (42, 43). In the present study, all patients undergoing root canal treatment received irrigation using a side-vented 30 G NaviTip needle, which was inserted 2 mm short of the working length in an attempt to minimize the irrigant extrusion, as reported elsewhere in the literature (44, 45). Intracanal cryotherapy was effective in reducing postoperative pain in this study.

Long-term follow-up studies in future research will be instrumental in advancing our understanding of the effects of intracanal cryotherapy on periradicular lesions.

In the present study, performance bias was minimized by randomizing the study groups after the root canal preparation. A limitation was that the operator's blinding was not possible because of the cooled syringes.

## 5 Conclusion

Cryotherapy has been shown to significantly alleviate postoperative pain following single-visit root canal treatment in molar teeth with pulp necrosis and symptomatic apical periodontitis. Considering these findings, we propose cryotherapy as a biocompatible, economical, and straightforward method for managing postoperative pain in single-visit root canal procedures.

## Data Availability

The raw data supporting the conclusions of this article will be made available by the authors, without undue reservation.
